# The Essential Phosphoinositide Kinase MSS-4 Is Required for Polar Hyphal Morphogenesis, Localizing to Sites of Growth and Cell Fusion in *Neurospora crassa*

**DOI:** 10.1371/journal.pone.0051454

**Published:** 2012-12-13

**Authors:** Anette Mähs, Till Ischebeck, Yvonne Heilig, Irene Stenzel, Franziska Hempel, Stephan Seiler, Ingo Heilmann

**Affiliations:** 1 Department of Plant Biochemistry, Albrecht-von-Haller-Institute for Plant Sciences, Georg-August-University Göttingen, Göttingen, Germany; 2 Institute for Microbiology and Genetics, Georg-August-University Göttingen, Göttingen, Germany; Cinvestav, Mexico

## Abstract

Fungal hyphae and plant pollen tubes are among the most highly polarized cells known and pose extraordinary requirements on their cell polarity machinery. Cellular morphogenesis is driven through the phospholipid-dependent organization at the apical plasma membrane. We characterized the contribution of phosphoinositides (PIs) in hyphal growth of the filamentous ascomycete *Neurospora crassa*. *MSS-4* is an essential gene and its deletion resulted in spherically growing cells that ultimately lyse. Two conditional *mss-4-*mutants exhibited altered hyphal morphology and aberrant branching at restrictive conditions that were complemented by expression of wild type *MSS-4*. Recombinant MSS-4 was characterized as a phosphatidylinositolmonophosphate-kinase phosphorylating phosphatidylinositol 4-phosphate (PtdIns4P) to phosphatidylinositol 4,5-bisphosphate (PtdIns(4,5)P_2_). PtdIns3P was also used as a substrate. Sequencing of two conditional *mss-4* alleles identified a single substitution of a highly conserved Y750 to N. The biochemical characterization of recombinant protein variants revealed Y750 as critical for PI4P 5-kinase activity of MSS-4 and of plant PI4P 5-kinases. The conditional growth defects of *mss-4* mutants were caused by severely reduced activity of MSS-4(Y750N), enabling the formation of only trace amounts of PtdIns(4,5)P_2_. In *N. crassa* hyphae, PtdIns(4,5)P_2_ localized predominantly in the plasma membrane of hyphae and along septa. Fluorescence-tagged MSS-4 formed a subapical collar at hyphal tips, localized to constricting septa and accumulated at contact points of fusing *N. crassa* germlings, indicating MSS-4 is responsible for the formation of relevant pools of PtdIns(4,5)P_2_ that control polar and directional growth and septation. *N. crassa* MSS-4 differs from yeast, plant and mammalian PI4P 5-kinases by containing additional protein domains. The N-terminal domain of *N. crassa* MSS-4 was required for correct membrane association. The data presented for *N. crassa* MSS-4 and its roles in hyphal growth are discussed with a comparative perspective on PI-control of polar tip growth in different organismic kingdoms.

## Introduction

Establishing polarity and maintaining cell shape are fundamental processes for cellular growth and the development of multicellular structures [1], [2]. Polar growth is mediated by polarized transport of vesicles to the apical plasma membrane of the cells. Membrane at the apex is expanded and extracellular components are secreted, while in subapical regions the cell recycles excess membranes. This cyclic movement of secretory vesicles and endosomes in polar growing cells is achieved by a complex protein machinery employing motor proteins and a dynamic actin-cytoskeleton controlled by regulatory small GTPases [3], [4], [5], [6]. Fungal hyphae share with neurons and pollen tubes the distinction of being amongst the most highly polarized cells known in biology [7], [8], [9]. Nevertheless, the relative impact of evolutionary conserved mechanisms and convergent traits in shaping these cell types is yet unresolved. This is partly a consequence of the fact that studies in different model systems have focused on different aspects of polar tip growth, and the available information has not been integrated to elucidate common as well as distinct features of cell polarity in plants, fungi and animals [1], [10], [11], [12].

In filamentous fungi, the hyphal tip contains sterol-rich membrane domains, which act as positional cues to determine a growing cell pole [6], [13], [14], [15], [16], likely by marking target areas for exocytotic vesicle tethering and fusion [17]. Together with other membrane lipids phosphoinositides (PIs) contribute to the formation of specialized membrane domains, which determine the polarized distribution of proteins in yeast [18], [19], animal [20] and plant cells [21], [22], [23], [24]. In particular, it has been demonstrated that phosphatidylinositol 4,5-bisphosphate (PtdIns(4,5)P_2_) localizes at the apices of tip-growing plant cells [25], [26], [27], [28] and in “shmoo-tips” of budding yeast cells treated with peptide mating pheromone [29]. PtdIns(4,5)P_2_ is formed by the phosphorylation of the lipid precursor, phosphatidylinositol 4-phosphate (PtdIns4P), a reaction catalyzed by phosphatidylinositol 4-phosphate 5-kinases (PI4P 5-kinases) [30], [31], [32]. Importantly, PI4P 5-kinases responsible for the formation of PtdIns(4,5)P_2_ display localization patterns that resemble the distribution of PtdIns(4,5)P_2_ in growing pollen tubes [33], [34], [35] and root hairs [36], [37], and equivalent localization patterns have been observed for budding yeast [18] and neurons [38], [39]. Membrane association of PI4P 5-kinases is thought to rely on recruitment through specific protein-protein interactions [40], [41]. Although well-studied and essential for polar growth in unicellular yeasts [42], we have currently no information about the role of PtdIns(4,5)P_2_ or the cellular distribution of PI4P5-kinases during hyphal growth in filament-forming fungi [6].

While it is assumed that PtdIns(4,5)P_2_-functions in filamentous fungi is similar to those in other eukaryotic models, several aspects complicate such a simplified view. First, in contrast to animals and plants where multiple isoforms of PI4P 5-kinases are common [30], [32], [43], the genomes of filamentous ascomycetes encode only one gene for a putative PI4P 5-kinase, *MSS-4* [32], [44]. For instance, in plant pollen tubes several PI4P 5-kinases produce PtdIns(4,5)P_2_ with distinct functionalities [33], [34], [35], [40], and such alternative functions would have to be performed by a single enzyme in filamentous fungi. A second aspect refers to possible alternative modes of action of *N. crassa* MSS-4, which might be mediated by specialized regulatory protein domains. *N. crassa* MSS-4 contains sequence extensions N- and C-terminal of the kinase domain, which distinguishes the enzyme from mammalian homologs. The additional protein domains of *N. crassa* MSS-4 are not similar in sequence to those of other PI4P 5-kinases and their functions remain unclear, resulting in a need to characterize the role of *N. crassa* MSS-4 in the control of polar tip growth as a base for further comparative studies.

Our working hypothesis was that apical localization of a functional enzyme capable of forming PtdIns(4,5)P_2_ in fungal hyphae contributes to the establishment of lipid microdomains guiding the machinery for polarized growth. Evidence for a role of *N. crassa MSS-4* in the control of polar tip growth derived from a large-scale screen for mutants defective in hyphal growth [45]. However, the function of the *mss-4* lesions has not been further characterized. In order to delineate the morphogenetic role of PtdIns(4,5)P_2_ in fungal hyphae, experiments with the predicted *N. crassa* phosphatidylinositolphosphate kinase MSS-4 were performed *in vivo* in *N. crassa* and plant pollen tubes as a heterologous expression system as well as *in vitro* on recombinant protein expressed in *E. coli*.

## Experimental Procedures

### Strains, Media and Growth Conditions for N. crassa

*N. crassa* strains used in this study are listed in Table 1 (also see [46]). General genetic procedures and media for *N. crassa* are available through the Fungal Genetics Stock Center (www.fgsc.net). An *mss-4* deletion strain (Δ*mss-4*) having the full-length open reading frame replaced by a hygromycin resistance cassette was generated by the *N. crassa* genome project [47] and was verified by Southern analysis. Growth rates of fungal strains were determined by measuring radius of colonies on agar plates starting with a well-established colony to exclude the lag phase of germination and the initial slow growth phase of a developing colony.

**Table 1:** *Neurospora crassa* strains used in this study.

Strain	Genotype	Source
*wild type 74*	*OR231 Mat A*	FGSC #987
*wild type ORS*	*SL6 Mat a*	FGSC #4200
*his-3 A*	*his-3 Mat A*	FGSC#6103
*his-3 a*	*his-3 Mat a*	FGSC #718
*mss-4(18-2)*	*mss-4(Y750N)*	[45]
*mss-4(34-10)*	*mss-4(Y750N)*	[45]
*mss-4(18-2)^compl^*	*mss-4(Y750N);mss-4(EC)*	This study
Δ*mss-4*	*mss-4*Δ*::hph^R^+mss-4^+^*	FGSC #15509
GFP-MSS4(1-1012)	*gfp-mss-4(1-1012)::his-3*	This study
GFP-MSS4(86-1012)	*gfp-mss-4(86-1012)::his-3*	This study

### Plasmid Construction and Expression of Tagged Proteins in N. crassa, Pollen Tubes and E. Coli

The ORF of *N. crassa mss-4* was annotated by the MIPS *N. crassa* database (http://mips.helmholtz-muenchen.de/genre/proj/ncrassa) and obtained by PCR from genomic DNA of wild type *N. crassa* using the oligonucleotide combination 5′-GAT CGA ATT CAT GAC CTC TTC GCC AAA TGG AG-3′ and 5′-GAT CGC GGC CGC TTG ACA CGC CTT GGG GCC GA-3′. The PCR-fragment was subcloned into the vector pGEM-Teasy (Promega). The resulting plasmid was designated *NcMSS4-pGEM-Teasy*. The insert was cloned into the vector *pENTR-EYFP* [34] as an *EcoRI-NotI* fragment and transferred by Gateway technology (Invitrogen) into the vector *pLatGW* (a kind gift from Dr. Wolfgang Dröge-Laser; Julius-Maximilians-University Würzburg, Germany) to generate the *mss-4-EYFP* fusion construct for expression in tobacco pollen tubes. Pollen tubes were transformed by particle bombardment and cultured as previously described [33]. For the expression of a GFP-MSS4 fusion protein in *N. crassa*, the *mss-4* ORF was amplified from *NcMSS4-pGEM-Teasy* using the oligonucleotide combination 5′-GAT CGG CGC GCC CAT GAC CTC TTC GCC AAA TGG AG-3′ and 5′-GAT CTC TAG ACT ATG ACA CGC CTT GGG GCC GA-3′ and transferred as an *XbaI*-Asc*I* fragment into pCCG1-N-GFP [48], creating the plasmid *pCCG1-N-GFP-MSS4(86-1012).* The extended N-terminus as annotated by the *N. crassa* BROAD database (http://www.broadinstitute.org/annotation/genome/neurospora/MultiHome.html) was amplified by PCR from genomic DNA of *N. crassa* with the oligonucleotide combination 5′-GAT CGG CGC GCC CAT GCC CTC CTT CGC ACC CGA-3′ and 5′-GAT CTA CGT AGC ATC GGA GCC GTC C-3′ and moved as *XbaI*-*Eco105I* fragment into *pCCG1-N-GFP-MSS4(86-1012)* to generate *pCCG1-N-GFP-MSS4(1-1012)*. For the expression of a GFP-HsPLCδ1-fusion protein in *N. crassa*, the ORF was amplified from *HsPLCδ1-pGEM-Teasy* [33] using the oligonucleotide combination 5′- GAT CGG CGC GCC CAT GGA TGA GGA TCT ACA GGC GC-3′ and 5′- GAT CTC TAG ACT AGA TCT TGT GCA GCC CCA GCA-3′ and transferred as an *XbaI*-Asc*I* fragment into pCCG1-N-GFP [48], creating the plasmid *pCCG1-N-GFP- HsPLCδ1. N. crassa* was transformed as previously described [49]. For the expression of *MSS-4* in *E. coli*, the ORF was amplified from *N. crassa* cDNA using the oligonucleotide combination 5′-GAT CCC ATG GGT ACC TCT TCG CCA AAT GGA G -3′ and 5′-GAT CCC ATG GCT GAC ACG CCT TGG GGC CGA -3′ and transferred as an *NcoI*-*NcoI* fragment into the expression plasmid *pETM-41* (EMBL Protein Expression and Purification Facility). *A. thaliana PIP5K2* was cloned into *pETM-41* as previously described [40]. Directed mutagenesis of the *MSS-4* (Y750N) and *PIP5K2* (Y738N) coding sequences was performed using the QuikChange Site-Directed Mutagenesis Kit (Stratagene) following the manufacturer’s recommendations and the oligonucleotide combinations *mss-4(Y750N)*, 5′-GCC CTC CCC CCT CAC GAA AAT GGC GAG CGG TTC ATC A-3′ and 5′-TGA TGA ACC GCT CGC CAT TTT CGT GAG GGG GGA GGG C -3′; *PIP5K2(Y738N)*, 5′-CTG CCG TTG ATC CCA AAC TAA ACT CAA GAA GGT TTA GAG AT-3′ and 5′-ATC TCT AAA CCT TCT TGA GTT TAG TTT GGG ATC AAC GGC AG-3′. Recombinant enzymes were expressed in *E. coli* strain BL21-AI (Invitrogen) at 25°C for 18 h after induction with 1 mM IPTG and 0.2% (w/v) L-arabinose. Cell lysates were obtained by sonification in a lysis buffer containing 50 mM Tris-HCl, 300 mM NaCl, 1 mM EDTA, and 10% (v/v) glycerol at pH 8.0.

### Lipid kinase Assays

Lipid kinase activity was assayed by monitoring the incorporation of radiolabel from γ[^32^P]ATP into defined lipid substrates (Avanti Polar Lipids) as described [50], using total extracts of BL-21-AI expression cultures. For assays using exogenous substrates, 5 µg of lipids were used. Each assay contained 10 µg of total bacterial protein, as determined by the Bradford method [51]. Recombinant expression levels were adjusted between individual cultures according to immunodetection of expressed MBP-tagged proteins. Radiolabeled lipid reaction products were separated by thin-layer-chromatography using silica S60 plates (Merck) and CHCl_3_:CH_3_OH:NH_4_OH:H_2_O (57∶50:4∶11, by volume) as a developing solvent [52], and visualized using a Fuji HLA-3000 phosphoimager. Substrate conversion was quantified by phosphoimager analysis using a standard curve of γ[^32^P]ATP for calibration of relative phosphate incorporation into lipids.

### Microscopy and Imaging

Low magnification documentation of fungal hyphae or colonies was performed as described [53] using an SZX16 stereomicroscope, equipped with a Colorview III camera and Cell^D^ imaging software (Olympus). Images were further processed using Photoshop CS2 (Adobe). Fluorescence microscopy with *N. crassa* was performed as described [54], [55]. An inverted Axio Observer. Z1 (Zeiss) microscope equipped with a QuantEM 512SC camera (Photometrics) and the slidebook 5.0 software (Intelligent Imaging Innovations) were used for image acquisition. Pollen tubes were recorded using either an Olympus BX51 epifluorescence microscope or a Zeiss LSM 510 confocal microscope. BX51: Images were obtained using an F41-028 HQ-Filterset for Yellow GFP (Olympus), an Olympus ColorView II camera and analySIS Docu 3.2 software (Soft-Imaging-Systems GmbH). LSM 510: EYFP was excited at 514 nm and imaged using an HFT 405/514/633 nm major beam splitter (MBS) and a 530–600 nm band pass filter. Fluorescence and transmitted light images were contrast-enhanced by adjusting brightness and γ-settings using image-processing software (Photoshop; Adobe Systems).

### Accession Numbers

Sequences used in this study can be identified by their accession numbers as follows: *AtPIP5K2*, At1g77740; *AtPIP5K5*, At2g41210; *NcMSS-4, NCU02295.*

## Results

### MSS-4 is Required for Cell Polarization and Polar Tip Extension in N. crassa

**Figure 1:**
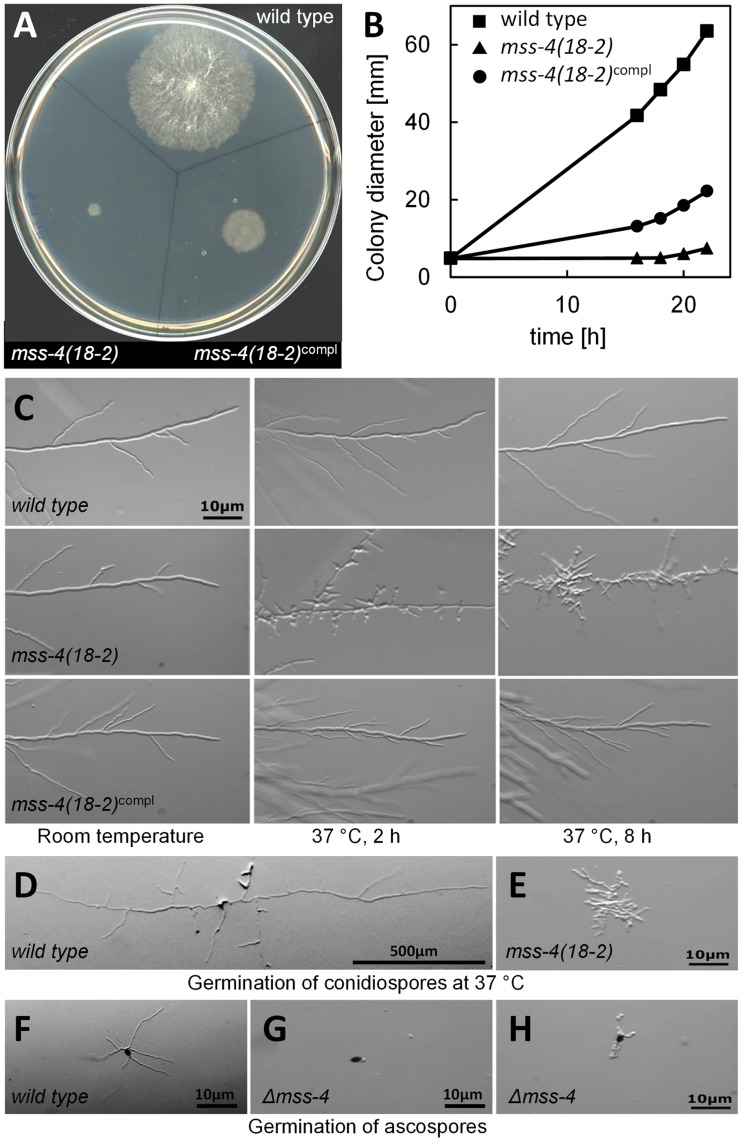
Phenotypic characterization of *N. crassa mss-4*. The growth of *N. crassa* wild type, the *mss-4(18-2)* mutant [45], and a complemented strain *mss-4(18-2)^compl^* ectopically expressing wild type MSS-4 on solid media was monitored by macroscopic examination after 24 h at 37°C (**A**). The increase in colony diameters of growing cultures over time was determined (**B**). Errors (SD) are too small to be seen at the scale given. The hyphal morphologies of wild type, the *mss-4(18-2)* mutant or *mss-4(18-2)^compl^* grown at room temperature or 2h and 8 h after shifting to 37°C, were microscopically characterized, as indicated (**C**). Germination of wild type (**D**) and *mss-4(18-2)* conidia was documented after 18 h at 37°C (**E**). Note the highly contorted and abnormal morphology of the *mss-4(18-2)*-strain. A strain carrying a deletion of the entire MSS-4 locus (Δ*mss-4*) failed to establish hyphae upon germination from ascospores (black) and, in contrast to wild type (**F**) was not viable (**G, H**). Experiments were repeated independently five times with similar results.

As basis for subsequent experiments, *N. crassa* wild type, *mss-4(18-2)* and the complemented mutant *mss-4(18-2)^compl^* were phenotypically characterized in detail. When grown at restrictive conditions of 37°C, wild type *N. crassa* formed colonies of approx. 60 mm in diameter within 24 h (Figure 1 A, B). In contrast, the temperature-sensitive mutant *mss-4(18-2)* did not exhibit substantial growth at 37°C (Figure 1 A, B). *mss-4(18-2)^compl^*, which contained an ectopic integration of the *MSS-4* ORF and 1 kb of 5′ and 3′ regulatory sequence, exhibited substantial growth. However, it was clearly slower than that of the wild type control (Figure 1 A, B). The hyphal morphology of *mss-4(18-2)* shifted to restrictive conditions was characterized by an abnormal cell shape, increased branching of hyphae, and subsequently cessation of tip extension and cell lysis (Figure 1 C). *mss-4(18-2)^compl^* exhibited only weakly increased branching of hyphae (Figure 1 C). *mss-4(18-2)* conidiospores germinating at 37°C displayed contorted and swollen cell morphology and increased branching, which resulted in highly restricted growth and the formation of compact colonies. *mss-4(18-2)* was still able to establish hyphae (Figure 1 D). In contrast, a *N. crassa* strain in which the full-length *MSS-4* ORF was replaced by a hygromycin resistance cassette (Δ*mss-4*) could only be maintained as a heterokaryon, having the mutation sheltered by the presence of nuclei containing a wild type copy of *mss-4*. Most uninucleate ascospores of Δ*mss-4* germinated only in an apolar manner (Figure 1 E), while those few that were able to establish germ tubes formed highly apolar and contorted growing cells that ultimately lysed (Figure 1 F), indicating that *mss-4* is an essential gene.

### N. crassa MSS-4 is a PI4P 5-kinase

**Figure 2:**
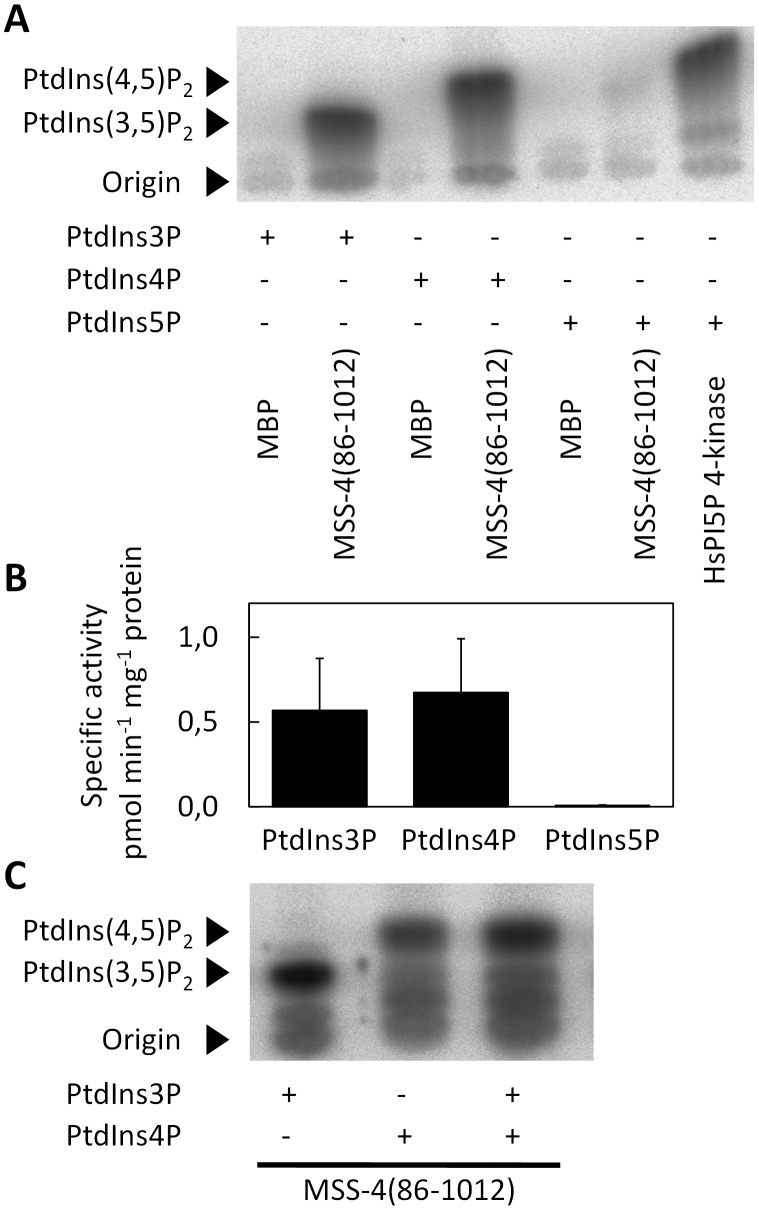
Biochemical characterization of recombinant MSS-4(86-1012) *in vitro*. MSS-4(86-1012) was heterologously expressed in *E. coli*. Recombinant extracts (10 µg of total protein) were incubated with 5 µg of lipid substrates in the presence of γ[^32^P]ATP. Phosphorylated lipids were extracted and separated by TLC. Images represent phosphoimager signals of radiolabeled lipids. Migration of lipid products was interpreted according to comigration with unlabeled authentic standards as previously described [71]. Phosphorylated lipid products were formed *in vitro* by MSS-4(86-1012) from different phosphatidylinositolmonophosphate-substrates, as indicated (**A**). Recombinant maltose-binding protein (MBP) served as a negative control; recombinant human PIP-kinase IIβ [56] served as a positive control for conversion of PtdIns5P. The phosphoimages presented in (**A**) were quantified (**B**). Specific activities were calculated using total protein content in the recombinant extracts. Data represent means ± SD of three independent experiments. The preference of MSS-4(86-1012) for PtdIns3P or PtdIns4P was tested in a competition experiment presenting lipids as individual substrates or as an equimolar mixture, as indicated (**C**). Phosphoimages are representative for at least three independent experiments.

In order to characterize the biochemical properties of MSS-4, we expressed recombinant protein in *E. coli* and then tested extracts *in vitro* for PI-kinase activity. In the course of a phylogenetic comparison of fungal, plant and animal PI4P 5-kinases [32], we had noted that the gene model presented for NCU02295/MSS-4 in the MIPS database might be ambiguous and that a possible alternative start codon exists 255 bp upstream of the originally proposed one. As *in vivo* data obtained later in the course of this study suggest the earlier start codon as the relevant one, all numbers identifying residues in MSS-4 in this study refer to the extended gene product of 1012 amino acids in length. Because MSS-4(1-1012) expressed poorly in *E. coli*, the biochemical characterization was carried out with MSS-4(86-1012), the variant originally proposed in the MIPS database. This approach is in line with previous reports, which confirm that PI4P 5-kinases do not require additional domains for catalytic activity and phospholipid specificity [34], [37], [40]. MSS-4(86-1012) was capable of phosphorylating both PtdIns3P and PtdIns4P to PtdIns(3,5)P_2_ and PtdIns(4,5)P_2_, respectively (Figure 2 A, B). PtdIns5P was not a substrate for MSS-4(86-1012), but was converted to PtdIns(4,5)P_2_ with high efficiency by a recombinant human PI5P 4-kinase [56] used as a positive control (Figure 2 A, B). When recombinant MSS-4(86-1012) was presented with equimolar amounts of PtdIns3P and PtdIns4P, the enzyme clearly preferred PtdIns4P, resulting in the predominant formation of PtdIns(4,5)P_2_ (Figure 2 C). These data indicate that the *N. crassa MSS-4*-gene encodes an active PI4P 5-kinase.

**Figure 3:**
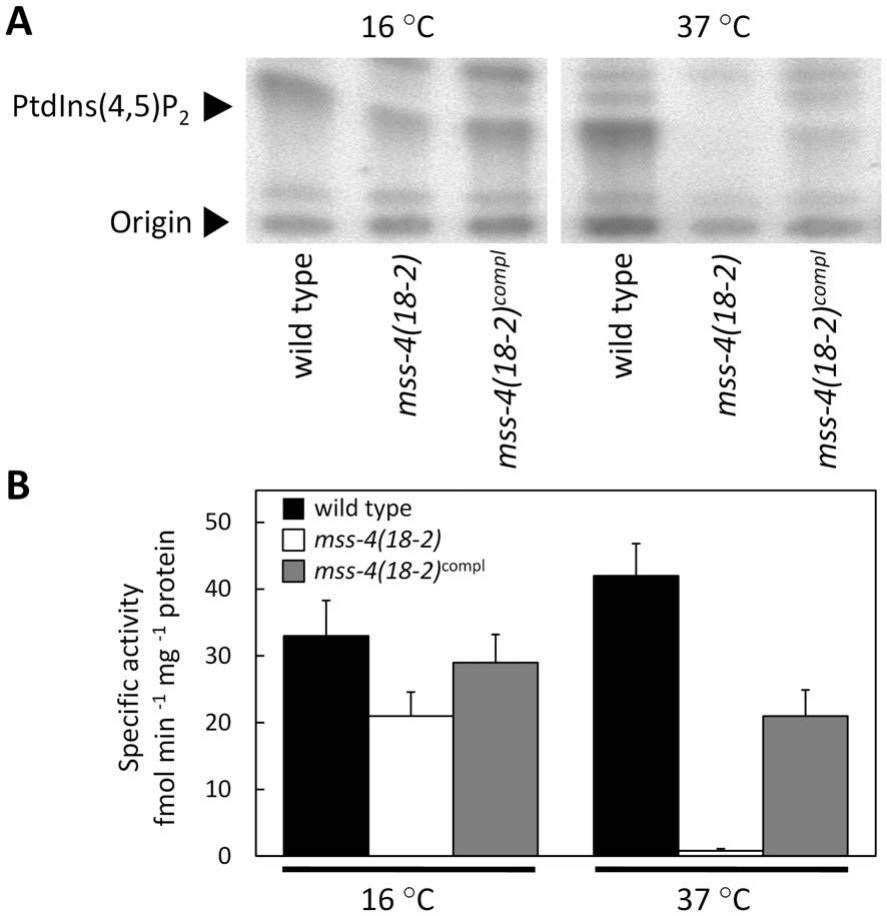
The *N. crassa mss-4* mutant has reduced PI4P 5-kinase activity at 37°C. The capability of membranes isolated from *N. crassa* wild type, *mss-4(18-2)* or mss-4(18-2)^compl^ grown at 16°C or at 37°C to phoshorylate PtdIns4P was determined. Membrane preparations containing 10 µg of total protein were incubated with 5 µg of lipid substrates in the presence of γ[^32^P]ATP. Phosphorylated lipids were extracted and separated by TLC. Images represent phosphoimager signals of radiolabeled lipids. Migration of lipid products was interpreted according to comigration with unlabeled authentic standards as previously described [71]. Formation of PtdIns(4,5)P_2_ in the indicated *N. crassa* strains (**A**). The image is representative for three independent experiments. Phosphoimages presented in (**A**) were quantified (**B**). Specific activities were calculated using total protein content in the recombinant extracts. Data represent means ± SD from three independent experiments.

Our work was based on the hypothesis that MSS-4 generates PtdIns(4,5)P_2_ required for polar tip growth of hyphae. To test this hypothesis, the intrinsic PI4P 5-kinase activity was determined in membranes purified from wild type, *mss-4(18-2)* and *mss-4(18-2)^compl^* grown at permissive and at restrictive conditions. While we detected PtdIns(4,5)P_2_ formation in membrane extracts of all three strains grown at 16°C, the *mss-4(18-2)* mutant was devoid of PI4P 5-kinase activity at 37°C (Figure 3 A). The data indicate that the cell polarity defects of *mss-4(18-2)* correlated with defective PI4P 5-kinase activity, and that ectopic expression of a genomic fragment encoding for wild type MSS-4 partially restored both hyphal growth and PI4P 5-kinase activity. Based on these observations, we conclude that the formation of PtdIns(4,5)P_2_ is necessary for cell polarization and tip growth in *N. crassa* and is mediated by MSS-4. Nevertheless, membranes purified from *mss-4(18-2)^compl^* grown at 37°C displayed substantial reduced PI4P 5-kinase activity compared the wild type control (21±7 versus 42±8 fmol min^−1^ mg^−1^ protein, respectively; n = 3). This may indicate that the ectopic integration of *MSS-4* may affect the expression level of the kinase. Alternatively, a dominant negative effect of the mutant MSS-4 variant is also possible.

### A highly Conserved Tyrosine is Required for the Function of Plant and Fungal PI4P 5-kinases

**Figure 4:**
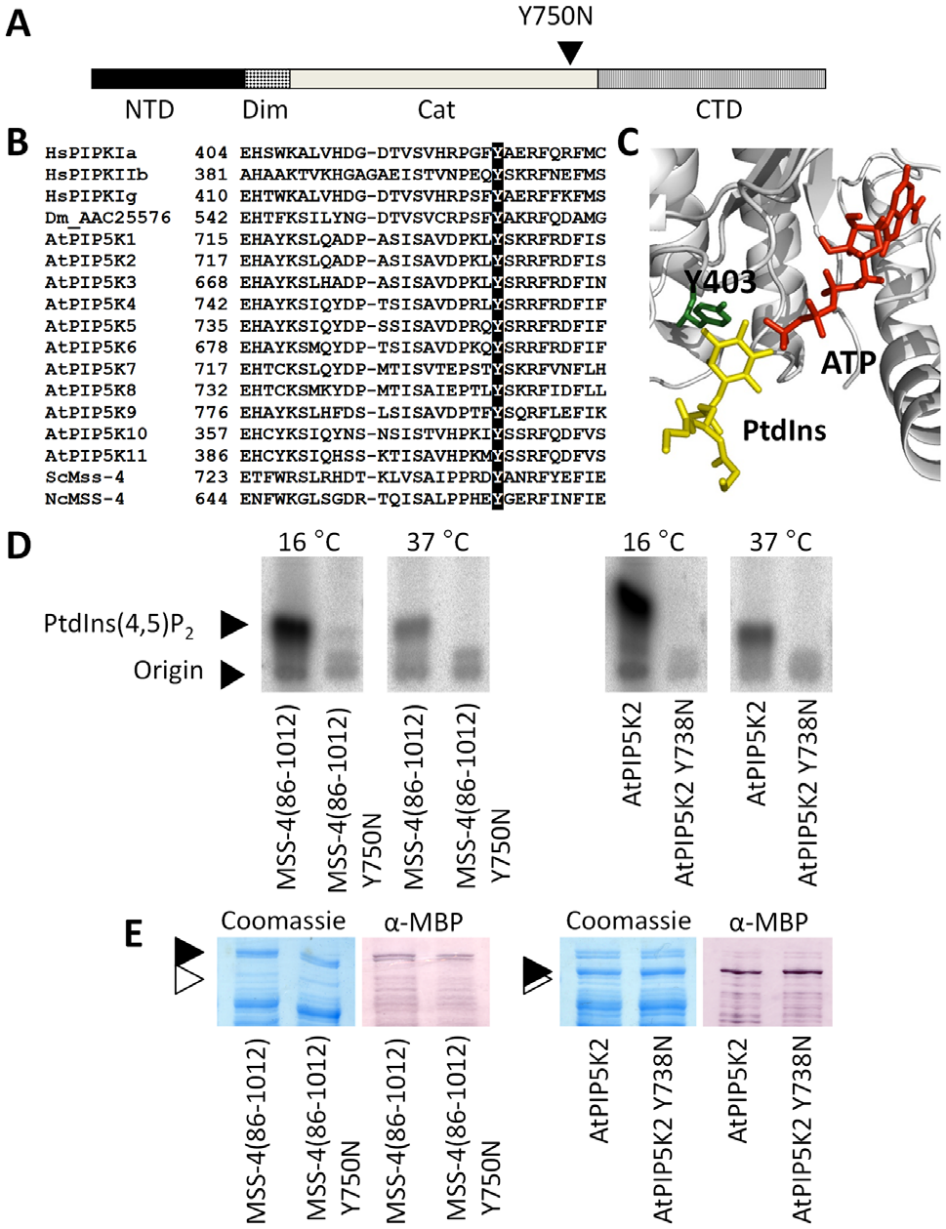
*mss-4* encodes an enzyme carrying a substitution of a conserved tyrosine residue for asparagine that results in reduced catalytic activity. The genomic loci of two *N. crassa mss-4* mutant strains were sequenced and the same nucleotide exchange 2248 T to A was found, resulting in an amino acid substitution from tyrosine 750 to asparagine. The domain structure of the deduced MSS-4 protein is presented in overview (**A**). The exchange Y750N is located in the C-terminal portion of the catalytic domain (arrowhead). NTD, N-terminal domains; Dim, Dimerization domain; Cat, catalytic domain; CTD, C-terminal domains. An alignment of partial amino acid sequences of various phosphatidylinositolmonophosphate kinases indicates that tyrosine 750 of NcMSS4 is strictly conserved in all sequences analyzed, including representatives from human, *Drosophila melanogaster*, *A. thaliana* and *S. cerevisiae* (**B**). According to the 3D-structure of the human PIP-kinase IIβ [56], the position of tyrosine 403 of the human enzyme, which corresponds to Y750 in MSS-4 from *N. crassa*, is in the substrate binding pocket (**C**). PtdIns, substrate analogon; ATP, position of ATP. Structure detail according to pdb entry 1bo1 [56]. Formation of PtdIns(4,5)P_2_ by MSS-4(86-1012) or MSS-4(86-1012;Y750N) was tested at 16°C or at 37°C, as indicated (**D**). Recombinant AtPIP5K2 carrying the corresponding exchange Y738N was tested *in vitro* for PtdIns(4,5)P_2_ for comparison. Wild type AtPIP5K2 was used as a positive control, as indicated. All activity tests were carried out using 10 µg of total bacterial protein and 5 µg of lipid substrate. (**E**) Expression of recombinant variants of MSS-4(86-1012) or AtPIP5K2 compared in (D) were tested by separating E. coli protein extracts by SDS-PAGE. Gels were stained with coomassie (left panels) or subjected to immunodetection using an anti-MBP antiserum (α-MBP; right panels), as indicated. Closed arrowheads indicate migration of MSS-4(86-1012), MSS-4(86-1012) Y750N, At-PIP5K2 or AtPIP5K2 Y738 N, as indicated. Open arrowheads indicate the migration of a 116 kDa size-marker. All experiments were performed three times with similar results.

The conditional phenotype of *mss-4(18-2)* suggested that the mutant encoded for a temperature-sensitive variant of MSS-4, which enabled growth at permissive temperature and precluded growth at prohibitive temperatures. Currently available conditional alleles encoding PI4P 5-kinases carry multiple point mutations, which are not readily applicable to enzymes from different biological sources. For instance, a temperature-sensitive variant of Mss4p from *Saccharomyces cerevisiae* [57] carries the changes Q351L, M392T, H532Q and K615R, of which only H532 is conserved in PI4P 5-kinases from plants, but none in human or animal sequences. A new temperature-sensitive PI4P 5-kinase allele would be a powerful tool to enable future studies on the roles of PtdIns(4,5)P_2_ in other biological systems. Therefore, we sequenced the genomic loci of *mss-4(18-2)* and of *mss-4(34-10)*, another previously identified conditional *mss-4* strain [45]. Both mutants contained only a single point mutation in nucleotide position 2248 (T to A) that resulted in the substitution of tyrosine 750 to asparagine (Figure 4). Position 750 of MSS-4 resides in the C-terminal portion of the catalytic domain (Figure 4 A). An alignment of MSS-4 with phosphoinositide kinases from human, *Drosophila melanogaster*, *A. thaliana* and *S. cerevisiae* (Figure 4 B) indicated that this tyrosine residue is strictly conserved among PI4P 5-kinases. To our knowledge, the invariant tyrosine residue has not been reported as critical for catalytic activity of PI4P 5-kinases. To determine if this conserved tyrosine might be part of the catalytic center of PI4P 5-kinases, the corresponding position of the human PIP-kinase IIβ (Y403) was highlighted in the available 3D-structure [56] (Figure 4 C). Based on this structural information, Y403 is located in immediate proximity of the substrate-binding pocket, opposing the position of the ATP co-substrate. When we assume that MSS-4 shares the overall organization of the catalytic center with the human PIP-kinase IIβ, the sequencing of two *mss-4* mutant loci identified a conserved tyrosine residue in the substrate-binding pocket as important for PI4P 5-kinase function.

To verify this notion, we analyzed the involvement of this conserved tyrosine in the temperature-sensitivity of *N. crassa* MSS-4 or other PI4P 5-kinases in more detail. PI4P 5-kinase activity of recombinant MSS-4(86-1012;Y750N) was compared with that of parental MSS-4(86-1012) at 16°C and at 37°C (Figure 4 D). Wild type MSS-4(86-1012) was active at 16°C and 37°C (658±78 and 232±21 fmol min^−1^ mg^−1^ protein; n = 3), whereas MSS-4(86-1012;Y750N) exhibited only trace activity at 16°C (15±5 fmol min^−1^ mg^−1^ protein; n = 3) and no detectable activity at 37°C.

Thus, the Y750N exchange resulted in drastically reduced catalytic activity rather than in temperature-dependent functionality of the mutated enzyme. In order to test whether the conserved tyrosine defined by the two *N. crassa mss-4* mutants was also important for the catalytic activity of PI4P 5-kinases from other organisms, an analogous variant of the *A. thaliana* PI4P 5-kinase was generated. When the PI4P 5-kinase activity of recombinant AtPIP5K2(Y738N) was compared with recombinant parental AtPIP5K2 at either 16°C or at 37°C, AtPIP5K2(Y738N) was found to be fully inactive at both temperatures (Figure 4 D), whereas wild type AtPIP5K2 was active at 16°C and at 37°C (759±95 and 332±12 fmol min^−1^ mg^−1^ protein, respectively; n = 3). Overall, these data indicate that the temperature-sensitive growth behavior of the *N. crassa mss-4* mutants is a result of minimal residual activity of MSS-4(Y750N), which seems sufficient to support growth at 16°C, but not at 37°C.

### N. crassa MSS-4 Localizes as a Subapical Membrane-associated Ring and Along Constricting Septa

**Figure 5:**
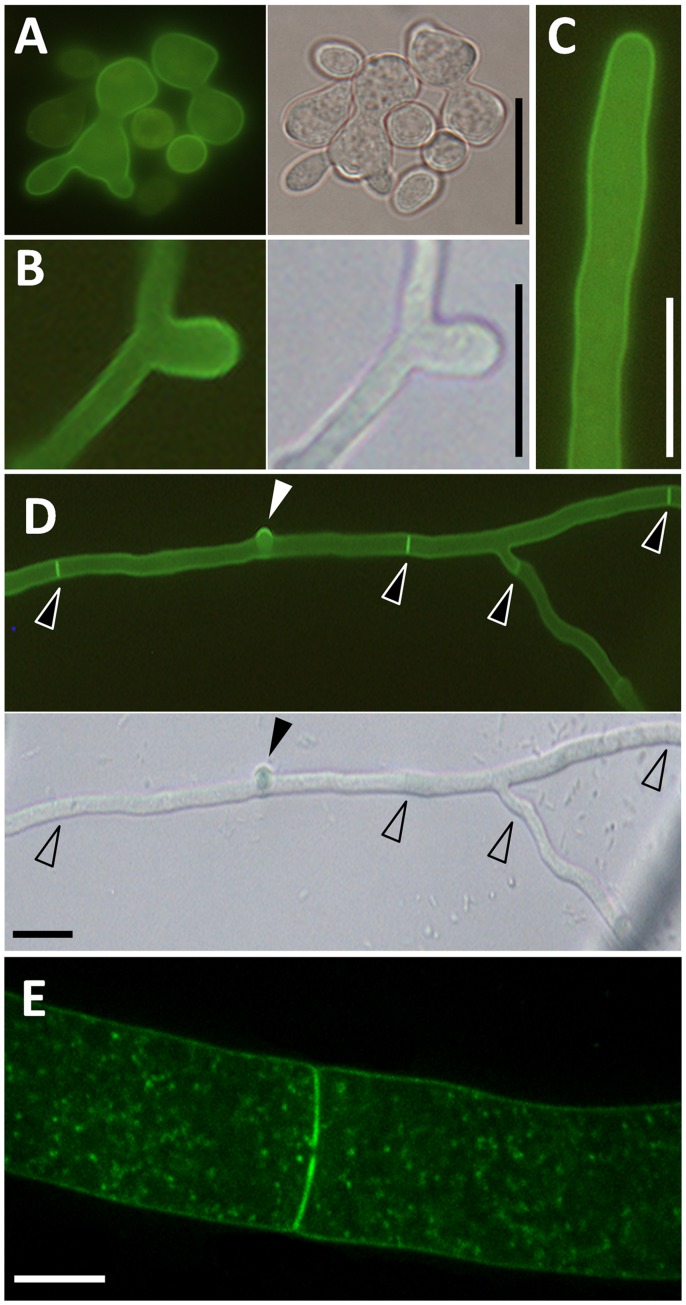
Distribution of a high-affinity fluorescent probe for PtdIns(4,5)P_2_ in *N. crassa* hyphae. The fluorescence distribution of PLCδ1-PH-EYFP was imaged in transgenic *N. crassa* hyphae. The reporter decorated the plasma membrane at different developmental stages, including germinating conidia (**A**), and accumulated at sites of hyphal branching or emergence (**B**). Reporter fluorescence was also present in the plasma membrane of hyphal tips (**C**). The reporter associated strongly with constricting septa (**D**). Decoration of septa (open arrowheads) and emerging hyphal tips (closed arrowhead) could be observed in the same cell (**D**). Overall plasma membrane association of the reporter, strong association with septa and cytosolic puncta were observed also in older, larger hyphae (**E**). Bars, 5 µm (A-C; F); 20 µm (E).

The data obtained so far suggested that MSS-4 regulates PtdIns(4,5)P_2_-levels required for apical tip growth. In line with this assumption, we determined the subcellular distribution of PtdIns(4,5)P_2_ *in vivo* using the specific reporter PLCδ1-PH-EYFP [33], [58]. In germinating conidia, the reporter fluorescence was associated with the plasma membrane (Figure 5 A). While fluorescence sometimes appeared stronger at emerging branches (Figure 5 B) or at hyphal apices (Figure 5 C), this pattern was not consistently observed, and reporter-fluorescence more often appeared evenly distributed along the entire plasma membrane. In addition to general plasma membrane association of the PtdIns(4,5)P_2_-reporter, PLCδ1-PH-EYFP appeared to stronger label emerging branches and constricting septa (Figure 5 D, E). In subapical regions of the hypha, the PtdIns(4,5)P_2_-reporter still associated with the plasma membrane and with septa and was also found associated with cytosolic puncta (Figure 5 E).

**Figure 6:**
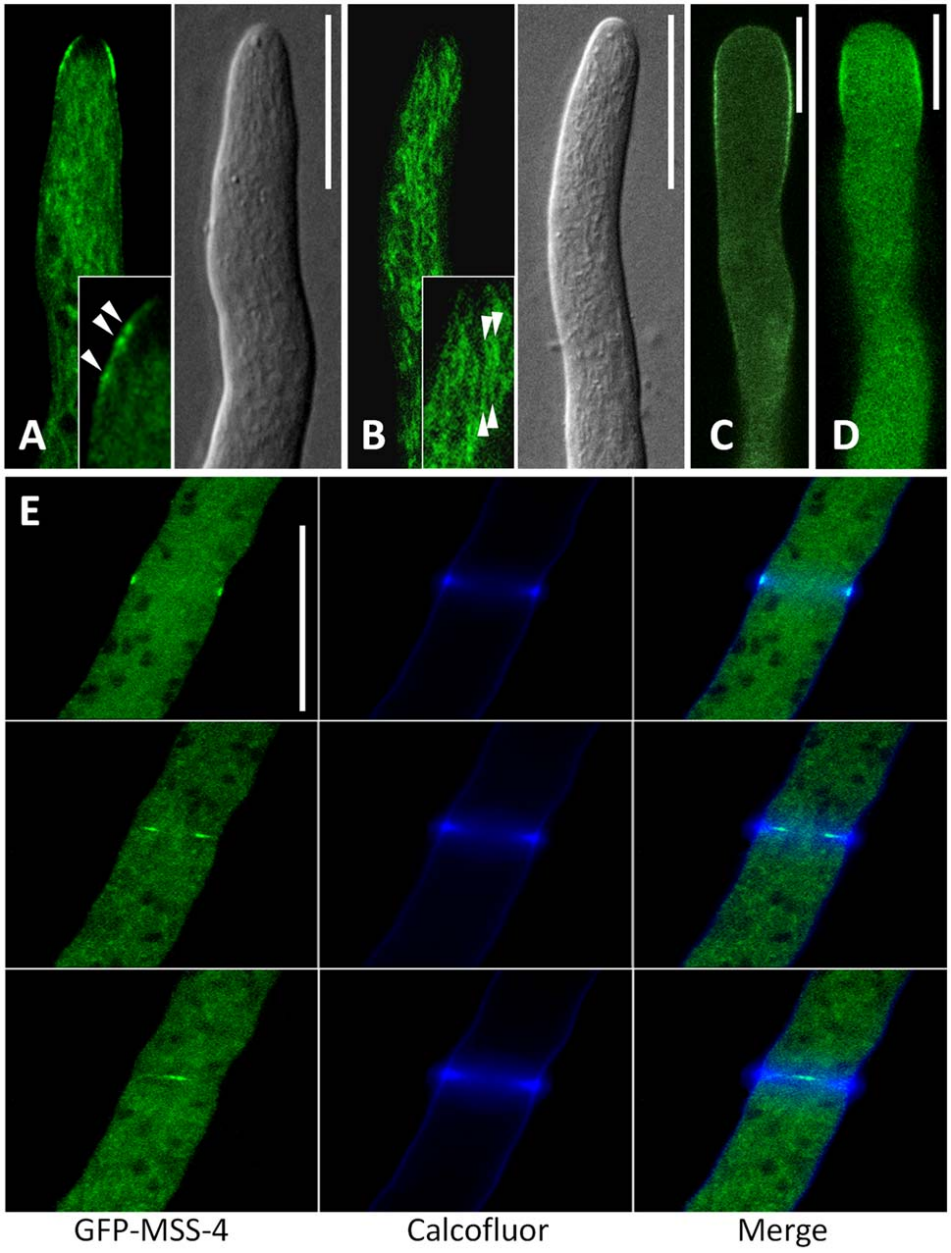
*N. crassa* MSS-4 localizes as a subapical membrane-associated ring and to constricting septa. MSS-4 fusion proteins carrying N- or C-terminal fluorescence-tags were expressed in *N. crassa* hyphae or in tobacco pollen tubes, and the fluorescence distribution was monitored by confocal microscopy. GFP-MSS-4 associated with a ring-like subapical plasma membrane domain in *N. crassa* hyphae (**A**). Plasma membrane association of GFP-MSS-4 was patchy (inset). Arrowheads indicate patches. Subapical membrane localization was reduced upon expression of MSS-4(86-1012)-EYFP (**B**) and displayed enhanced association with cytosolic filaments or endomembrane-structures (inset). Arrowheads indicate possible filaments. MSS-4-EYFP associated with a ring-like subapical plasma membrane domain in tobacco pollen tubes (**C**). AtPIP5K5:EYFP localized in a ring-like subapical plasma membrane domain in tobacco pollen tubes (**D**) and is shown for comparison. GFP-MSS-4 also decorated constricting septa (**E**). Left panels, GFP; mid panels, Calcofluor; right panels, merged images. Images are representative for >100 transgenic cells observed for each expression. Bars, 10 µm.

Reassured by the localization of PtdIns(4,5)P_2_ at the plasma membrane and its increased abundance at sites of active growth (i.e. septum and hyphal tip), we determined the subcellular localization of MSS-4 by expressing a GFP-MSS-4 fusion protein in *N. crassa*. GFP-MSS-4 localized in a ring-like membrane domain immediately behind the apex of growing hyphae (Figure 6 A). Plasma membrane localization appeared in spots rather than a continuous, smooth signal, possibly indicating association with membrane microdomains (insert in Figure 6 A). GFP-MSS-4 also decorated the plasma membrane in regions distal of the apical ring, but fluorescence was much weaker than at the apical domain. Moreover, we observed cytosolic fluorescence and association with cytosolic filaments or endomembranes of unknown nature in addition to the apical plasma membrane localization, indicative of a portion of the enzyme present in a soluble state and associated with intracellular structures (Figure 6 A). This observation is in line with previous reports on the dynamic subcellular distribution of PI4P 5-kinases, which peripherally associate with membranes [56]. Subapical plasma membrane association of PI4P 5-kinases of the subfamily B from *A. thaliana* requires the presence of N-terminal regulatory domains other than the catalytic domain [36], [37], [40]. As *N. crassa* MSS-4 can also be categorized as a PI4P 5-kinase of subfamily B [32] we tested whether a deletion of the first 85 amino acids, as proposed by the alternative gene model for MSS-4, would impair membrane localization of MSS-4. When GFP-MSS-4(86-1012) was expressed in *N. crassa*, the fusion protein accumulated primarily in the cytoplasm and associated with intracellular filaments and/or endomembranes (Figure 6 B). The plasma membrane localization was strongly reduced. Reciprocally, we also expressed two fragments of MSS-4 that contained the N-terminal region, but neither GFP-MSS-4(1-85) nor GFP-MSS-4(1-355) displayed membrane association or apical localization (data not shown). The failure of such smaller N-terminal fragments to localize has previously been reported from equivalent experiments using plant PI4P 5-kinases and might be a result of misfolding or the requirement of additional domains for proper membrane association of PI4P 5-kinases [40]. The requirement for the N-terminal domain (Figure 6 B) indicated similarities in the modes of plasma membrane recruitment of *N. crassa* MSS-4 with *A. thaliana* PI4P 5-kinases [36], [37], [40]. To further substantiate this notion, *N. crassa* MSS-4-EYFP was transiently expressed in tobacco pollen tubes (Figure 6 C). The observed subapical membrane association of the heterologously expressed kinase was similar to the localization in the native fungal context, but also patterns reported for plant PI4P 5-kinases from *A. thaliana* [33], [35] or tobacco [40] (Figure 6 D, *A. thaliana* PIP5K5 as an example). The data indicate that localization of PI4P 5-kinases, such as MSS-4 and its plant homologs as a subapical membrane-associated collar is conserved across kingdoms and can be found in plant pollen tubes and root hairs as well as in fungal hyphae.

**Figure 7:**
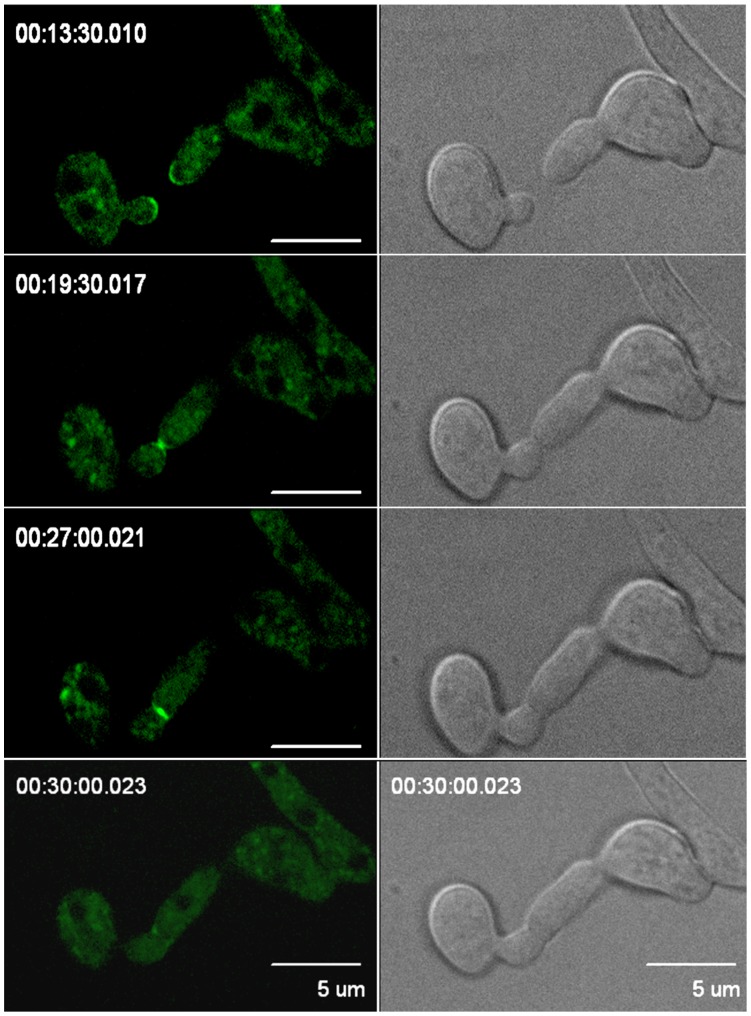
Localization of GFP-MSS-4 in tips of communicating cells during chemotropic growth and cell fusion. Cells expressing GFP-MSS-4 were imaged in a time-lapse experiment spanning 30 min. Selected time points are shown, as indicated. Left panels, GFP; right panels, bright field. Note the increased accumulation of GFP-MSS-4 at the contact point and during generation of the fusion pore. Images are representative for 20 fusion events observed. Bars, 5 µm.

Besides similarities of GFP-MSS-4 localization in tip growing cells, some hyphal-specific localization patterns were observed. Fungal septa represent cell walls and associated plasma membrane that partition growing hyphae into smaller cytosolic sections. GFP-MSS-4 localized to sites initiating septum formation, decorating the inner edges of the constricting ring of cell wall (Figure 6 E; Movie S1). Another example for a process known in filamentous ascomycetes, but not for tip-growing plant cells, is intercellular communication and subsequent fusion to establish a mycelial network [59], [60], [61], [62]. *N. crassa* germling fusion involves the fusion of specialized cell protrusions that have been termed conidial anastomosis tubes (CATs). CATs are characterized by positive tropic growth. When cell communication of *N. crassa* CATs was investigated, GFP-MSS-4 was strongly enriched at both tips of mutually attracting CATs that concentrated at the contact sites of two fusing cells (Figure 7;. Movie S2). Moreover, upon initiation of cell fusion, the accumulated GFP-MSS-4 expanded concentrically around the expanding fusion pore. Together, these localization patterns observed for GFP-MSS-4 in *N. crassa* suggest a central role for PtdIns(4,5)P_2_-formation at sites of polar growth at the hyphal apex and constricting septa. During hyphal fusion MSS-4 might contribute to membrane rearrangements required for the establishment of cytosolic contacts between fusing cells.

## Discussion

In order to delineate what perturbations of PI-metabolism might contribute to the phenotypes of *mss-4(18-2)* and Δ*mss-4*, the biochemical properties of MSS-4 must be considered. When PtdIns3P or PtdIns4P were tested individually as substrates for recombinant MSS-4(86-1012), the enzyme converted both substrates equally well. This contrasts the situation of plant PI4P 5-kinases, which exhibit high specificity towards PtdIns4P [33], [34], [37], [52], [63]. PtdIns5P was not a substrate for MSS-4(86-1012), in line with the presence of a glutamate residue in position 723, which is implicated in specifying PI4P 5-kinases [64]. The notion that MSS-4(86-1012) can produce PtdIns(3,5)P_2_ or PtdIns(4,5)P_2_ from PtdIns3P and PtdIns4P, respectively, indicates a low degree of substrate discrimination during the production of PtdIns-bisphosphates *in vitro*. Therefore, we cannot rule out that PtdIns(3,5)P_2_, PtdIns(4,5)P_2_, or both lipids are formed by MSS-4 *in vivo* and that both lipids might be required for hyphal growth. However, a functional role for PtdIns(4,5)P_2_ rather than PtdIns(3,5)P_2_ is supported by the observations that (i) PtdIns4P was preferentially used in *in vitro* competition experiments, resulting in the formation of PtdIns(4,5)P_2_, (ii) membrane fractions purified from *mss-4(18-2)* growth at 37°C displayed drastically reduced PI4P 5-kinase activity, and (iii) growth defects manifested despite the presence of a putative PI3P 5-kinase encoded by the locus NCU02083, which is similar to the yeast PI3P 5-kinase, Fab1p, [32] and likely capable of generating PtdIns(3,5)P_2_ in the *mss-4(18-2)* background. Thus, our data strongly suggest that the reduced formation of PtdIns(4,5)P_2_ is the reason for the cell polarity defects of the *N. crassa mss-4* mutants.

The conditional *N. crassa mss-4* strains allowed the identification of an invariant tyrosine, which is critical for catalytic activity of MSS-4 and related PI4P 5-kinases. Mapping of the homologous Y403 in the 3D-structure of the human PIP-kinase IIβ [56] places this residue in close proximity to the substrate-binding pocket within the active site. The position of this conserved Y750 suggests that it is likely involved in catalysis by binding or orienting the substrate in MSS-4. An involvement in catalysis rather than conditional activity of this substitution is also consistent with the fact that a conditional MSS-4 variant in *S. cerevisiae* contained multiple, non-conserved amino acid substitutions [57]. The conclusion is also in line with our finding of close-to-normal PtdIns(4,5)P_2_ levels in extracts of *mss-4(18-2)* grown at 16°C, while PtdIns(4,5)P_2_ at 37°C was drastically reduced. In summary, these data suggest that poor catalytic activity of MSS-4(Y750N) in combination with increased turnover of PtdIns(4,5)P_2_ at 37°C may be the cause for the observed cell polarity defects in *mss-4(18-2)*.

The morphological defects observed in the two *mss-4* mutants partially resemble phenotypic characteristics of mutant plant root hairs [36], [37] or pollen tubes [33], [35], [65]. For instance, pollen tubes of *A. thaliana pip5k6* single or *pip5k4 pip5k5* and *pip5k10 pip5k11* double mutants exhibit impaired germination, reduced tip expansion and increased sensitivity to the actin depolymerizing drug, latrunculin B [33], [34], [35], [66]. Since plasma membrane domains containing PtdIns(4,5)P_2_ as well as other lipids, such as sphingolipids or sterols [15], [16] define the sites of polarized expansion, the functionality of such membrane domains, and thus, polar tip extension are compromised if one or more lipid constituents are missing. The increased branching rates of *N. crassa mss-4(18-2)* may be a consequence of mislocalized plasma membrane domains, leading to recruitment of the machinery for cell expansion to multiple sites rather than one apical domain as in wild type. Alternatively, increased hyphal branching in *mss-4(18-2)* might be a result of altered biophysical properties of the cell wall or effects on intracellular signaling [32].

PtdIns(4,5)P_2_ was associated with the plasma membrane in *N. crassa* hyphae. Reporter fluorescence was found along the entire plasma membrane and was, thus, different from that previously observed in yeast challenged by mating pheromone [29]. Increased reporter-fluorescence at new branching sites (cf. Figure 5 D) was not consistently observed. The distribution of PtdIns(4,5)P_2_ resembled that of MSS-4, which was also associated with sites of active growth, with the notable exception that PtdIns(4,5)P_2_ was detected also in the extreme apex of the cells (cf. Figure 5 C), whereas MSS-4 was abesnt from the very apex (cf. Figure 6 A). A possible explanation for the extended localization of PtdIns(4,5)P_2_ is that it diffuses laterally from sites of its biogenesis either as a free lipid or bound to target proteins in or at the membrane. The subcellular distribution of MSS-4 in the hyphal context closely resembles patterns reported for PI4P 5-kinases in plant pollen tubes. The membrane domain decorated by MSS-4 in *N. crassa* hyphae corresponds to the zone of endocytosis in fungal hyphae [67], [68], [69] and in pollen tubes [70]. Expression of GFP-MSS-4(86-1012) resulted in loss of subapical plasma membrane association and accumulation in the cytosol and at undefined endomembranes, indicating a role for the N-terminal 85 amino acids in mediating membrane recruitment of MSS-4. Interestingly, both variants of MSS-4 (MSS-4(1-1012) and MSS-4(86-1012)) complemented the *mss-4(18-2)* defects (data not shown). At this point it remains unclear whether MSS-4(1-1012) and MSS-4(86-1012) represent alternative gene products of *MSS-4* that differ in their subcellular distribution, possibly as part of mechanisms that control subcellular levels of PtdIns(4,5)P_2_. Alternatively, complementation of nonfunctional MSS-4(86-1012) in *mss-4(18-2)* might be the result of heterodimerization with endogenous MSS-4(Y750N) and correct localization by a piggyback mechanism.

Recently, a non-conserved N-terminal domain (NTD) was characterized as essential for membrane association of plant PI4P 5-kinases of subfamily B [40]. The similar localization patterns of native and heterologously expressed *N. crassa* MSS-4-EYFP and plant PI4P 5-kinases [33], [35], [37] in tobacco pollen tubes suggest a similar mechanism for controlling membrane recruitment of *N. crassa* MSS-4(1-1012) and plant PI4P 5-kinases via N-terminal protein domains. It has previously been proposed that PI4P 5-kinases are recruited to their target membranes by protein-protein interactions, possibly through their N-terminal domains. While N-terminal domains of *N. crassa* MSS-4 and plant PI4P 5-kinases are not similar in sequence, they may nonetheless enable interaction with other partners of a conserved recruitment machinery in the respective heterologous system. Alternatively, it is possible that heterodimerization of MSS-4 with plant-endogenous PI4P 5-kinases recruited the *N. crassa* enzyme to the apical plasma membrane in tobacco pollen tubes.

Overall, the data presented in this study indicate that tip growth in the filamentous ascomycete *N. crassa* requires the formation of PtdIns(4,5)P_2_ by the PI4P 5-kinase MSS-4 at the hyphal tip and, possibly, during septation to support polar growth. The patterns of MSS-4 localization in living *N. crassa* cells suggest a mode of action for PtdIns(4,5)P_2_ that is similar to that proposed for polar growing cells from other biological models, such as plant pollen tubes or root hairs. Structural similarities between plant root hairs or pollen tubes and fungal hyphae are, thus, accompanied by similarities in regulatory membrane lipids contributing to the control of polar tip growth.

## Supporting Information

Movie S1 **Time-course of GFP-MSS-4 localization during septum formation.** GFP-MSS-4 formed cortical rings at incipient septation sites that constricted during septum formation and accumulated around the septal pore of the completed septum. The plasma membrane was stained with FM4-64. Images were captured at 45s intervals. (**A**) GFP channel; (**B**) FM4-64 channel; (**C**) merged.
(MOV)

Movie S2 **Time-course of GFP-MSS-4 localization during tropic growth of two communicating cells.** GFP-MSS-4 localized as a membrane-associated cap to both tips and strongly accumulated at the contact point after physical contact of both cells and during generation of the fusion pore. Images were captured at 45 s intervals. (**A**) GFP channel; (**B**) phase contrast.
(MOV)
